# Hepatoid Adenocarcinoma of the Urachus

**DOI:** 10.1155/2016/1871807

**Published:** 2016-10-10

**Authors:** Daniel Fernando Gallego, Carlos Muñoz, Carlos Andrés Jimenez, Edwin Carrascal

**Affiliations:** ^1^Department of Pathology, University of Washington, Seattle, WA, USA; ^2^Department of Surgery, Mercy Medical Center, Baltimore, MD, USA; ^3^Department of Pathology, Fundacion Valle del Lili, Cali, Colombia; ^4^Department of Pathology, Universidad del Valle, Cali, Colombia

## Abstract

Hepatoid adenocarcinoma of the urachus is a rare condition. We present the case of a 51-year-old female who developed abdominal pain and hematuria. Pelvic magnetic resonance imaging (MRI) reported an urachal mass with invasion to the bladder that was resected by partial cystectomy. On light microscopy the tumor resembled liver architecture, with polygonal atypical cells in nest formation and trabecular structures. Immunochemistry was positive for alfa-fetoprotein (AFP) and serum AFP was elevated. Hepatoid adenocarcinomas have been reported in multiple organs, being most commonly found in the stomach and the ovaries. Bladder compromise has been rarely described in the literature, and it has been associated with poor prognosis, low remission rates, and early metastasis.

## 1. Introduction

Hepatoid adenocarcinoma (HAC) is an extrahepatic tumor that is morphologically similar to the architecture of hepatocellular carcinoma (HCC) [1]. On light microscopy, it is composed of large polygonal cells in a nest pattern and occasionally exhibits bile canaliculi formation producing bile pigment [2]. On immunohistochemistry hepatoid adenocarcinomas could be positive for alfa-fetoprotein (AFP), epithelial membrane antigen (EMA), and albumin [3]. Hepatocyte paraffin-1 (HepPar1), glypican-3, and arginase 1 are useful hepatic markers and canalicular patterns can react with polyclonal anticarcinoembryonic antigen (CEA). Elevated serum AFP has been correlated as well with HAC [1, 4], but it is not always present [5]. Clinically patients are usually older males presenting with hematuria [6] and tumors can be very aggressive presenting with lung metastases [2]. HAC have been reported in multiple organs but are most commonly found in the stomach and the ovaries [7]. We report the clinicopathological features of a patient with a HAC of the urachus with invasion to the bladder.

## 2. Case Report

Our patient is a 51-year-old Latin American female, who presented with a 6-month history of mild generalized abdominal pain and one episode of gross hematuria. On physical examination there was a distended abdomen and pain on deep palpation.

**Figure 1:**
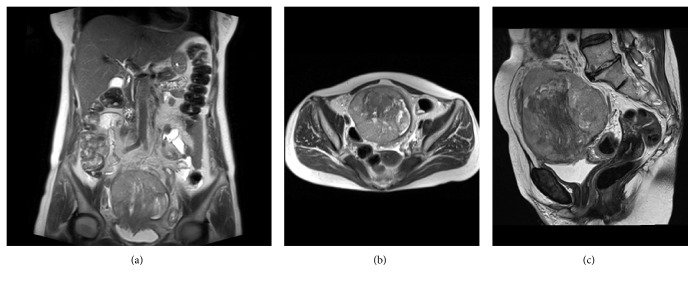
Abdominopelvic Magnetic Resonance Image (MRI) showing a 12 × 11 × 10 cm mass with necrotic center and heterogeneous signal on T2-weighted sequence. Coronal (a), axial (b), and sagittal (c) MRI images revealed the mass located at the umbilicus extending to the bladder dome, which is highly suggestive of urachal cancer.

Initial investigations included a transvaginal ultrasound, which revealed a pelvic tumor extending towards the abdominal cavity. Pelvic MRI showed a mass located in the bladder dome that measured 12 × 10 × 11 cm, with heterogenous signal in T2 and a necrotic center (Figure 1). The exophytic appearance and the mass location were suggestive of a lesion originated in the urachal diverticulum. The mass compromised other structures such as fat and muscle around the bladder, as well as a portion of the ileum, which showed thickening and narrowing of its lumen. Retroperitoneal and iliac lymph nodes were increased in size. No lesions were evident on the bladder neck, trigonum vesicae, or ureters.

Subsequently, the patient underwent a transurethral resection (TUR) for a microscopic diagnosis. The initial pathology reported limited sample with insufficient material for diagnosis. Due to the tumor characteristics with evident invasion to other tissues, it was considered appropriate to perform a palliative surgery, consistent in partial cystectomy plus partial ileum resection.

**Figure 2:**
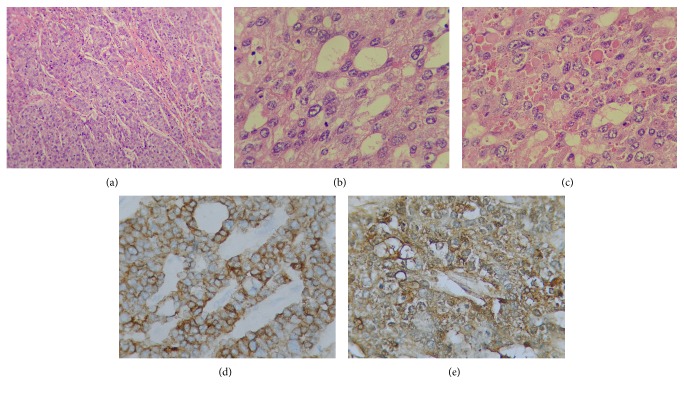
Hepatoid adenocarcinoma of the urachus. (a) Trabecular pattern (H&E ×100). (b) Close-up view of hepatoid cells showing pleomorphism, prominent nucleoli, and (c) intracytoplasmic hyaline globules (b and c, H&E ×400). (d) Positive immunostain for Pan-Keratin (AE1/AE3) and (e) intracytoplasmic positive cells for alfa-fetoprotein (AFP).

The pathology report from the partial cystectomy showed an ulcerated and hemorrhagic lesion measuring 11 × 9 × 7 cm macroscopically. Light microscopy revealed a neoplastic proliferation of large polygonal epithelial cells, arranged in a nesting and trabecular pattern, with central necrosis. These cells showed focal pleomorphism, prominent nucleoli, wide eosinophilic cytoplasm, and occasional hyaline PAS positive globules (Figure 2). More than ten mitoses in ten high power fields were found. The neoplasm showed extensive involvement of the serosa with ileum wall involvement. There was no vascular or neural invasion. However, three lymph nodes were compromised.

Immunohistochemical study showed cytoplasmic positivity in the tumor cells against AFP and Pan-Keratin (AE1/AE3) (Figure 2), with a 70% Ki67 proliferation index. There was no expression of CK20, CK5/6, Ck7, EMA, CEA, S100, synaptophysin, chromogranin, enolase, TTF-1, progesterone/estrogen receptors, PLAP, and CD56.

All the previous findings were consistent with invasive high-grade hepatoid adenocarcinoma of the bladder originated in the urachus. Stage was T3a N2, with free surgical borders.

After surgery, the patient remained stable, with symptomatic improvement and no further complications. A subsequent abdominal Computer Axial Tomography (CAT) scan ruled out the possibility of a metastatic hepatocellular carcinoma or other abdominal lesions. Serum AFP was elevated.

After four months of surgery, the patient has been treated with three cycles of chemotherapy, with Gemcitabine and Cisplatin, with adequate response and no adverse effects. Serum AFP level at this time is significantly lower compared to previous measurements.

## 3. Discussion

Hepatoid adenocarcinoma (HAC) is any epithelial cancer from a nonliver origin, which resembles hepatic cells morphology [1]. One of the most important steps for diagnosis is to rule out extrahepatic metastases of HCC. HAC commonly involve organs such as the stomach and ovaries [7]. In addition, involvement of the lungs, gallbladder, pancreas, uterus, and adrenals has been reported [1, 7–11]. The most common site of metastasis is the lung [2].

In our case the tumor originated from the urachus involving the bladder. This location is very rare. Urachal tumors represent 0,01% of adult cancers and 0,17 to 0,34% of bladder cancers [12, 13]. Since 1994, 9 cases of HAC of the bladder have been reported [2, 4–6, 14, 15] and this constitutes the second case of hepatoid urachal adenocarcinoma reported in the literature [16].

In Table 1 we summarize the demographic and clinical data of 10 cases of HAC of the bladder, including our case. According to the details provided, the most common manifestation was hematuria in 8 cases. The average age at presentation was 68 years and 6 of the cases were males. Four patients were staged T3 and 3 patients had metastatic disease at diagnosis. The size of the tumor of our patient was 11 × 9 cm, bigger than the previously reported cases [4].

Our case is a high-grade/high-stage tumor, different from the usual behavior described of low-grade even in high-stage cases [2, 5, 6].

On immunohistochemistry, AFP was positive and CEA was negative, as originally described by Prat et al. in hepatoid ovary tumors [17]. We used monoclonal anti-CEA that resulted in being negative, and although positive staining would support the diagnosis due to its high specificity, the result obtained does not rule it out [18]. Other markers as chromogranin and NSE were negative as expected [2, 5].

Our patient had a Ki67 proliferation index of 70%. Ki 67 has been characterized as a reliable indicator of muscle invasion and useful in confirmation of high-grade lesions in certain tumors [19]. In our review, a case of a high-grade HAC of the stomach showed elevated Ki67 [20]. However, we need a bigger sample of cases to make a correlation between Ki67 proliferation index and staging in hepatoid adenocarcinomas.

This type of tumor has a poor prognosis and low cure rates. The longest survival after diagnosis has been 26 months [2], which has been inversely associated with staging [4, 6]. All the patients with metastatic disease at diagnosis have died by the time of the case report [2].

Treatment for HAC in other organs has been characterized [9], but not in the bladder. We consider that the role of chemotherapy requires further investigation, especially in the field of drug resistance expression of ATP Binding Cassette (ABC) and drug transporters in hepatoid adenocarcinomas [21].

## 4. Conclusions

In summary, we present a case of hepatoid adenocarcinoma of the urachus with involvement of the bladder. The patient presented with abdominal pain, gross hematuria, and a bladder mass. On light microscopy the lesion resembled the histology of hepatocellular carcinoma. On immunochemistry AFP was positive and serum AFP was elevated.

To our knowledge this is the second case of hepatoid adenocarcinoma of the urachus reported in literature and first case reported in Latin America.

## Figures and Tables

**Table 1:** Demographic and clinical data of reported cases.

Case number/reference	Age/sex	Symptoms	Tumor size (cm)	Serum AFP	Therapy	Stage	Follow-up	Status
(1) Sinard et al. [14]	68/F	Hydronephrosis	2.5	N/A	TUR	T3a	17 months	AWD
(2) Yamada et al. [4]	89/F	Hematuria	6.5 × 5.5	+	TC	T2b	1 month	Unknown
(3) Burgués et al. [5]	71/M	Hematuria	N/A	WNL	TUR	T2	N/A	AWD
(4) Lopez-Beltran et al. [2]	66/M	Hematuria	6.5	+	TC	T3a	14 months	Metastasis, DOD
(5) Lopez-Beltran et al. [2]	85/M	Hematuria	80 g	N/A	TUR	T2	12 months	Metastasis, DOD
(6) Lopez-Beltran et al. [2]	61/M	Hematuria	5 × 5	+	TC	T3a	19 months	Metastasis, DOD
(7) Lopez-Beltran et al. [2]	68/M	Hematuria	1.5	+	TUR	T1	26 months	NED
(8) Kawamura et al. [15]	79/M	Hematuria	1	+	TUR	Ta	19 months	NED
(9) Sekino et al. [6]	49/F	No symptoms	0.6	N/A	TUR	T1	20 months	NED
(10) Present case	51/F	Hematuria	11 × 9	+	PC	T3a	4 months	AWD

AFP: alfa-fetoprotein, AWD: alive with disease, DOD: died of disease, N/A: not available, NED: no evidence of disease, PC: partial cystectomy, TC: total cystectomy, TUR: transurethral resection, and WNL: within normal limits.
